# An assessment of the simulated performance of basic clinical procedures by junior doctors during the first year of clinical practice

**DOI:** 10.1186/s12909-023-04545-1

**Published:** 2023-08-09

**Authors:** Paul O’Connor, Ambyr Reid, Orla Mongan, Cara Egan, Bronwyn Reid-McDermott, Philip Parackal Augusthinose, Michael Smith, Ruth Cooney, Dara Byrne

**Affiliations:** 1 Department of General Practice, School of Medicine, University of Galway, 1 Distillery Road, Newcastle, Co Galway, Galway, H91 TK33 Ireland; 2 Irish Centre for Applied Patient Safety and Simulation, University of Galway, Galway, Ireland; 3 School of Medicine, University of Galway, Galway, Ireland; 4 Saolta University Health Care Group, Galway, Ireland

**Keywords:** Confidence, Proficiency, Clinical procedures, Junior doctors

## Abstract

**Background:**

Upon entering the healthcare system, junior doctors may lack the skills required to care for patients, and feel unprepared for their role, with considerable variation in the level of proficiency in the performance of particular clinical procedures.

**Objective:**

To compare the performance and proficiency (self-report and observed) of the performance of nine basic clinical procedures.

**Methods:**

Seventeen interns were observed performing nine clinical procedures in a simulated setting in June 2021 (Assessment 1) and January 2022 (Assessment 2). The observers identified whether each step in the procedure was performed correctly, and provided an overall assessment of proficiency. The participants also rated their own level proficiency.

**Results:**

At Assessment 1 the number of steps performed correctly ranged from a mean of 41.9–83.5%. At Assessment 2 the number of steps performed correctly ranged from a mean of 41.9–97.8%. The most common median proficiency rating for Assessment 1 was ‘close supervision’, and was ‘indirect supervision’ at Assessment 2. There was a significant and large effect size in the improvement in performance from Assessment 1 to Assessment 2. Low correlations were found between observer and self-reported proficiency in performance of the procedures.

**Conclusions:**

The large improvement in performance across the two assessments is encouraging. However, there is a need to address the variability in performance on graduation from medical school, and to ensure that any assessment of proficiency is not only reliant on self-report.

## Introduction

Upon entering the healthcare system, many junior doctors lack the skills required to care for patients, and feel unprepared for their role [1–3]. There can also be considerable variation between these new medical school graduates in the level of competency in the performance of basic clinical procedures [4]. This variability is because the experiences of junior doctors differs depending on the setting in which they are placed, and the level of supervision and support that they receive during their clinical training [5, 6]. It is also important to acknowledge that junior doctors do not all necessarily acquire the skills required to deliver care to patients at the same rate, with differing level of practice required before competence is achieved [5]. Traditionally the early years of postgraduate medical training have been based upon a time-based apprenticeship model. Under this model of education, junior doctors advance to the next stage of training not based upon whether they have developed the necessary competencies, but as a result of the time they have spent in the role.

In the Republic of Ireland (RoI), every new medical school graduate completes one year of internship. The goal of internship is to provide new medical school graduates with education, training, and clinical responsibility in the real healthcare environment. However, as is the case in many other countries, there is no summative assessment of performance of this first period of postgraduate training. Therefore, it is unknown as to whether a newly graduated doctor has acquired the competences required to advance to the next stage of training. It was found that after six months of working as an intern, the vast majority (> 80%) reported that they could execute basic clinical procedures without the need for direct supervision [4]. However, this determination was based upon self-reported proficiency- rather than through independent assessment.

Determining competency based upon self-report is attractive as it requires little resources to complete. However, it has consistently been found that in medicine, as well as other domains, that self-report competency is not consistent with observed competency [7, 8]. A systematic review of studies examining the accuracy of doctors’ self-assessed competence as compared to observed measurement concluded that, in the majority of the studies, doctors do not accurately self-assess their own competence [7]. Moreover, in a number of studies in this review, it was found that the least skilled doctors tended to be the most confident in their abilities- a phenomenon consistent with the Dunning-Kruger effect [8].

The research question to be answer by our study was: what is the observed, and self-reported, proficiency of newly graduated medical students in completing nine basic clinical procedures immediately on graduation from medical school, and seven months into the intern year? Performance of the procedures were assessed in a simulated environment. The rationale for the assessment after seven months is that this is the time point during internship at which most interns believe they can complete these procedures without the need for direct supervision [4]. It was hypothesised that there will be an improvement in observed, and self-reported proficiency, in all nine of the clinical procedures at the second assessment as compared to the first assessment.

## Methods

### Participants

Interns from the West/Northwest (WNW) Intern Training Network (ITN) in the RoI.

### Context

Internship is the first year of postgraduate clinical practice for doctors in the RoI. Each intern is trained in one of six national ITN. The intern rotates through four clinical attachments, each of three months in duration.

### Selection of clinical procedures

Nine clinical procedures were identified for assessment- see Table 1. These clinical procedures were taken from the Irish Entrustable Professional Attributes (EPA) framework [9, 10].

**Table 1:** Clinical procedures and expected proficiency

Clinical Procedure	Expected proficiency….	
	on graduating medical school*	on completing internship^#^
Electrocardiogram (ECG)	Indirect supervision (4)**	Indirect supervision (4)
Blood sampling & blood cultures from a central line & tunnelled lines	Not stated	Indirect supervision (4)
Peripheral intravenous cannulation	Direct supervision (2)	Indirect supervision (4)
Preparation, reconstitution, dilution & administration of IV drugs	Not stated	Indirect supervision (4)
Arterial blood gas sampling	Direct supervision (2)	Indirect supervision (4)
Nasogastric tube insertion	Direct supervision (2)	Indirect supervision (4)
Urinary catheter insertion	Direct supervision (2)	Indirect supervision (4)
Venepuncture	Indirect supervision (4)	Indirect supervision (4)
Blood cultures from a peripheral vein	Direct supervision (2)	Indirect supervision (4)

*from the Irish EPA framework [9, 10]

^#^ from the UK GMC [11]

** Number in brackets represents the point on the scale for the level of proficiency

Interns are expected to be able to complete these procedures with indirect supervision (see Table 2 for a definition) by the end of internship [9]. The Irish EPA framework provides no guidance for expectations of the proficiency of performance of these procedures on graduation from medical school. However, the UK General Medical Council (GMC) [11] has delineated the level of proficiency expected for newly graduated medical school graduates in seven of these nine procedures (see Tables 1 and 2).

**Table 2:** Levels of proficiency

Level	Proficiency	Supervision	Equivalent GMC level of competence
1	Intern has acquired relevant knowledge and skills, but not enough to perform the activity.	Pre-practice	
2	Intern may perform an activity under direct supervision, with supervisor in the same room, deciding the intensity of supervision required.	Close supervision	Safe to practice in a simulator (1)/ direct supervision (2)
3	The intern may perform an activity with direct, intermittent supervision: the intern asks for supervision as required.	Intermittent supervision	
4	The intern may perform an activity independently with mainly informal, indirect supervision.	Indirect supervision	Indirect supervision (3)
5	Intern may provide supervision and instruction to junior learners.	Experienced intern	

### Ethical approval

Ethical approval was received from Galway University Hospital ethics board on the 20th October 2020 (reference: CA 2241).

### Study design

**Fig. 1:**
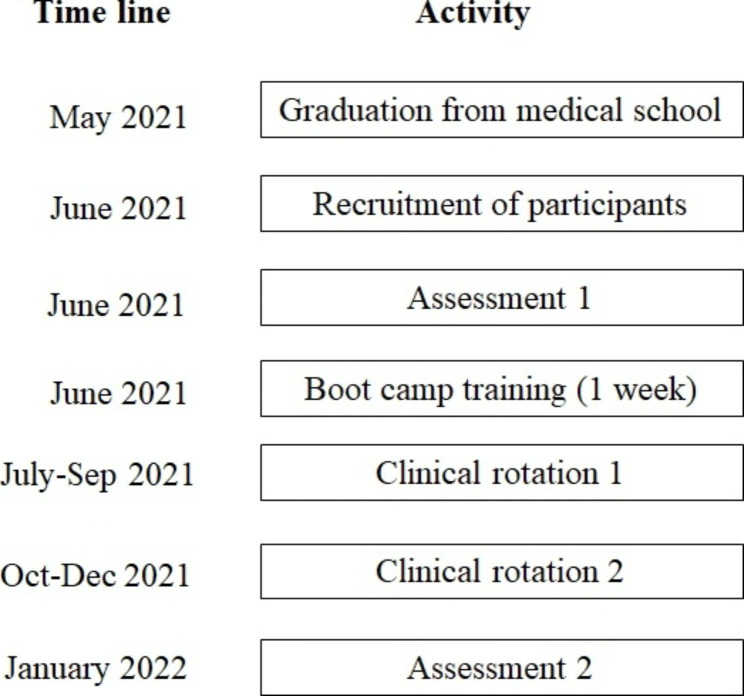
Research project timeline

The study utilised a repeated measures design. An overview of the timeline of the research project is shown in Fig. 1.

### Sample size calculation

Previous research suggests that a very large effect size could be expected in this study. To illustrate, Lydon et al. [12] carried out a study in which junior paediatric trainees were trained to carry out lumbar puncture using a simulator. At baseline the participants performed 31.8% (st dev = 13.3) of the steps correctly, and at a month or more after completion the training the participants performed 95.7% (st dev = 7.7) of the steps correctly. This is an effect size, as measured by Cohen’s d, of 5.9.

To calculate the sample size for our study, *a priori* sample size calculation was completed using GPower 3.1 with effect size of 1.0, power of 0.95 and a level of significance of 5% (two-tailed), for matched pairs of participants using the Wilcoxon Signed Rank test. This power analysis identified a need to recruit 16 participants. However, 22 participants were recruited in order to allow for some expected attrition of participants across the two assessments.

### Recruitment

Recruitment took place in June 2021 prior to the ‘boot camp’ training that occurs between completing medical school and starting internship in the WNW intern training network (see Fig. 1). Interns from the intern training network were made aware of the research by email, and the first 22 newly graduated medical students who agreed to participate in the study were recruited as participants. The participants provided signed informed consent, and were given a 100 Euro voucher for each assessment session in which they participated.

### Procedure

The performance of the nine procedures outlined in Table 1 were assessed in a simulated setting immediately prior to boot camp in June 2021 (Assessment 1; see Fig. 1) in a simulated setting. The assessment was repeated in January 2022 (Assessment 2; see Fig. 1). The second assessment was carried out at the end of their second internship rotation. All of the participants had completed a surgical and medical rotation before Assessment 2.

#### Self-assessment

Before the participants carried out the formal assessment, they complete a clinical procedures self-assessment questionnaire in which they were asked to provide a global rating of their competence to perform each of the nine clinical skills being assessed (see Table 1) using the proficiency scale outlined in Table 2. The scale is the same as recommended for assessing entrustability in the Irish EPA framework [9, 10]. In addition, at Assessment 2 only, the participants completed another questionnaire asking them how often they had completed each of the clinical procedures during their last rotation from 0 (never) to 5 (every shift).

#### Observer assessment

After completing the self-assessment questionnaire, the interns rotated through a simulated assessment station for each of the nine clinical procedures. Every station had the consumables and part-task simulator required to complete the procedure. Participants were randomly assigned to an initial station, and rotated to the next station after 25 minutes- whether or not the procedure was completed. The rationale for each station lasting 25 minutes was that this was considered by the subject matter experts to be a sufficiently generous amount of time in which the longest procedure should be easily completed.

The participants were evaluated by assessors who were knowledgeable about the procedure under observation. Each assessment station had one assessor. The assessors observed the intern perform the procedure and recorded whether or not each step in the procedure was performed correctly or incorrectly. The steps in the procedures were derived through a combination of the clinical experience of subject matter experts, and a review of existing guidelines and protocols. After the assessor had completed the checklist assessment they also gave a global rating using the proficiency scale described in Table 2.

### Formal training received by the participants between assessment 1 and assessment 2

Formal training was provided in all of the nine clinical procedures by the intern training network during the week long ‘boot camp’ delivered immediately after Assessment 1 (see Fig. 1)- with the exception of nasogastric tube insertion and male catheterisation. Formal training in these two procedures was provided during one of the weekly mandatory intern training sessions completed during the first three months of internship. In the training to perform the nine clinical procedures the interns are provided with the checklist and all of the consumables (e.g. gloves) required to complete the procedure. Interns receive 30–45 minutes of supervised practice in each of the procedures. The interns then carry out the procedure, at least once, on a part-task simulator with feedback and tuition provided by an instructor.

The interns receive no other further formal training in how to perform the procedures. However, they receive informal instruction in the clinical environment when the procedures are performed on patients. The interns also had access to documents describing the steps require to complete each procedure, and narrated videos showing the procedures being performed by the intern training network.

### Analysis

The data was analysed using IBM SPSS for Windows (version 22). If a participant only completed Assessment 1, then their data was removed from the analysis. If a participant failed to complete both assessments for one of the procedures, then a pairwise deletion was applied. A Wilcoxon sign rank test was used to statistically compare the number of steps performed correctly, the observer evaluation of proficiency, and the self-report proficiency at the two time points. Cohen’s *d* was used to measure the effect size of the difference. A commonly used interpretation of this statistic is to refer to an effect size as small (*d* = 0.2), medium (*d* = 0.5), or large (*d* = 0.8)[13]. Spearman’s rho was used to calculate the correlation between the percentage of steps performed correctly, the observer global rating, and the participant competency rating.

## Results

### Participants

A total of 17 intern (10 men and 7 women) participants completed Assessments 1 and 2. The participants were all graduates from medical schools in RoI. There were five additional interns that only completed Assessment 1, so their data was removed from the analysis. All of the procedures were completed by all 17 participants on both occasions, except two interns did not complete urinary catheter insertion at Assessment 2 as they had to return to work.

### Observations of steps in the clinical procedure

Table 3 provides a summary of the mean percentage of steps that were performed correctly in each clinical procedure. A significant greater percentage of the steps were performed correctly for all of the procedures at Assessment 2 as compared to Assessment 1 with the exception of: blood sampling and blood cultures from central line and tunnelled line preparation; and reconstitution, dilution, and administration of IV drugs (see Table 3). There was a large effect size in the difference in performance for all of the procedures except for blood sampling and blood cultures from central line and tunnelled line preparation. It is also noteworthy that the standard deviation in the percentage of correct steps decreased for all procedures from Assessment 1 to Assessment 2.

**Table 3:** Percentage of steps performed correctly in each procedure at Assessment 1 and Assessment 2

	Assessment 1		Assessment 2		
Clinical procedure	Mean	St Dev	Mean	St Dev	Effect size (*d*)
Electrocardiogram	77.8	21.0	94.2	5.3	1.07*
Blood sampling & blood cultures from central line & tunnelled line	83.5	18.3	94.9	6.0	0.83*
Peripheral intravenous cannulation	72.4	22.6	89.4	8.1	1.00*
Prep, reconstitution, dilution & admin of IV drugs	53.5	23.5	58.3	13.3	0.25
Arterial blood gas sampling	41.9	26.8	86.0	6.6	2.26*
Nasogastric tube insertion	49.0	19.9	85.8	7.7	2.44*
Urinary catheter insertion^#^	51.9	35.6	97.8	3.4	1.81*
Venepuncture	53.5	16.6	68.5	10.9	1.07*
Blood cultures from a peripheral vein	52.5	21.9	70.6	11.0	1.05*

^#^ 15 participants completed this procedure at Assessment 2

* significant difference between Assessment 1 and Assessment 2 at p < .01

### Observer assessed proficiency

Table 4 shows the median proficiency rating provided by the assessors, the interquartile range, and the percentage of participants that reached the desired level of proficiency stated by the UK GMC [11] on completion of medical school at Assessment 1, and ‘indirect supervision’ (level 4 or higher) at Assessment 2. There was a significant increase in the rating of proficiency for all of the procedures- with the exception of preparation, reconstitution, dilution, and administration of IV drugs.

**Table 4:** Summary of observer assessed proficiency rating for each procedure

	Median (IQR)		% meet or exceed GMC standard	**% ≥4 proficiency**	
	**Assessment**		**Assessment**	Assessment	
Clinical procedure	1	2	1	1	2
Electrocardiogram	2 (1)	5 (0)*	17.6	17.6	100
Blood sampling & blood cultures from central & tunnelled lines	2 (1)	4 (1)*	-	17.6	58.8
Peripheral intravenous cannulation	2 (1)	4 (1)*	100	17.6	94.1
Prep, reconstitution, dilution & admin of IV drugs	2 (1)	2 (1)	-	0	5.9
Arterial blood gas sampling	2 (1)	3 (1)*	64.7	5.9	41.2
Nasogastric tube insertion	3 (1)	4 (1)*	100	5.9	70.6
Urinary catheter insertion^#^	2 (2)	5 (1)*	70.6	11.8	100
Venepuncture	2 (1)	4 (1)*	11.8	11.8	64.7
Blood cultures from a peripheral vein	2 (1)	4 (0)*	100	5.9	82.4

*significant difference between Assessment 1 and Assessment 2 at p < .01

### Self-assessed proficiency

Table 5 shows the self-reported median proficiency rating, interquartile range, the percentage of participants that believed they had reached the equivalent level of proficiency stated by the UK GMC [11] on completion of medical school at Assessment 1, the percentage of participants that reached proficiency of ‘indirect supervision’ (level 4 or higher) at Assessment 2, and the percentage of participants that reported carrying out the procedure at least once a week. There was a significant increase in the rating of the proficiency for all of the procedures.

**Table 5:** Summary of self-assessed proficiency rating and frequency of performance of each procedure

	Median (IQR)		% meet or exceed GMC standard	**% ≥4 proficiency**		% perform min once a week
	**Assessment**		**Assessment**	Assessment		
Clinical procedure	1	2	1	1	2	
Electrocardiogram	3 (2)	5 (0)*	29.4	29.4	100	78.6
Blood sampling & blood cultures from central & tunnelled lines	1 (1)	4 (1)*	-	0	82.4	28.6
Peripheral intravenous cannulation	1 (1)	5 (0)*	41.2	0	100	100
Prep, reconstitution, dilution, & admin of IV drugs	1 (1)	2 (1)*	-	0	23.5	21.4
Arterial blood gas sampling	2 (1)	5 (1)*	52.9	0	94.1	21.4
Nasogastric tube insertion	2 (2)	4 (1)*	58.8	23.5	88.2	7.1
Urinary catheter insertion	1 (1)	4 (1)*	47.1	0	76.5	21.4
Venepuncture	3 (2)	5 (0)*	29.4	29.4	100	100
Blood cultures from a peripheral vein	2 (0)	5 (0)*	82.4	0	100	71.4

*significant difference between Assessment 1 and Assessment 2 at p < .01

^#^ Three participant did not provided this data

### Correlation between observer and self-ratings of proficiency

Table 6 shows the correlations between the percentage of steps performed correctly, observer assessment, self-assessment at assessments 1 and 2. It can be seen that there is an increase in the correlation between observer and self-ratings of proficiency at Assessment 2 as compared to Assessment 1.

**Table 6:** Correlations between the percentage of steps performed correctly, observer assessment, self-assessment at Assessments 1 and 2

	Assessment 1			Assessment 2		
	% of steps correct	Observer assessment	Self assessment	% of steps correct	Observer assessment	Self assessment
**% of steps correct**	1.0	0.65**	0.08	1.0	0.22**	-0.11
**Observer assessment**	0.65**	1.0	0.17*	0.22**	1.0	0.42**
**Self assessment**	0.08	0.17*	1.0	-0.11	0.42**	1.0

*significant at p < .05

**significant at p < .01

## Discussion

Medical training has traditionally been delivered using a time-based apprenticeship model. As such, doctors advance to the next stages of training based on time, and not upon an assessment of proficiency and readiness. The aim of this study was to evaluate the performance of nine basic clinical procedures on graduation from medical school, and then after seven months into the intern training year.

For the majority of the assessed clinical procedures there was a significant and large improvement across the two assessments. This finding is consistent with a survey of interns in the RoI that found that the majority of interns believed that they only required indirect supervision in these nine procedures after six months of internship [4]. However, there were three procedures for which the observers judged that more than half of the participants required more than indirect supervision: preparation, reconstitution, dilution, and administration of IV drugs; arterial blood gas sampling; and nasogastric tube insertion. Considering that the participants reported that these procedures were infrequently performed in the clinical environment, this is unsurprising. For final year medical student, it has been found that there is a correlation between the number of times a basic clinical procedure is performed and confidence [14]. Therefore, it is recommended that interns are given opportunities to practice these three procedures as part of the formal intern training on a number of occasions during the intern year, as it appear that they are not getting the opportunity to develop the skills required to perform these procedures in the clinical environment.

The majority of the participants were judged to require only indirect supervision for six of the procedures at the second assessment. This demonstrates that the combination of formal teaching, informal teaching on the ward, and clinical experience allows interns to reach the desired level of proficiency before the end of the intern year of training. However, there may still be a cost to patient care during this period of learning in terms of a negative impact upon the efficiency of the health service resulting from a need to repeat procedures if the first attempts are unsuccessful [15], ordering unnecessary tests [16], as well as negatively impacting the psychological well-being to the interns themselves from failing to perform a procedure successfully [17]. These are all potential issues that could be addressed through increased practice and assessment during medical school.

There was considerable variability in the ability of the participants to perform the nine procedural skills on graduation from medical school. Moreover, there were only two procedures for which the independent raters thought that all of the participants had met or exceeded the proficiency recommendations outlined by the UK GMC [11]. This finding is consistent with other studies that have found that high percentages of newly graduated medical students report feeling under-prepared to begin working in a hospital, [18] and variability in their confidence to perform specific clinical procedures [1] in the few weeks between finishing medical school and starting as a junior doctor in the hospital [19]. It is suggested that there is a need to ensure that these procedural skills are taught, and assessed, in medical school where there is more time for teaching, assessment, and remediation as compared to the month between graduating medical school and starting work as an intern. There is also a need to establish an agreed level of proficiency that should be achieved on completion of medical school in the RoI- and not only the end of internship.

It has been suggested that simulation-based assessment is an approach to addressing the challenges of reliably conducting assessment in a busy clinical environment [10]. However, irrespective of the method, the main barrier is the resources required to perform the assessment. It may be tempting to use self-reported assessment as this is easy to perform. However, consistent with other literature [7, 8], our study found low correlation between observer and self-reported proficiency. Interestingly, the correlation been the observer and self-reported proficiency was higher at the second assessment. This may suggest that the experience of attempting to carry out the procedure in the clinical environment allowed the participants to more accurately assess their own proficiency. Approaches to assessment must be affordable, practical, effective and cost-effective, acceptability to all stakeholders (junior doctors, supervisors, and patients), do not result in unexpected side-effects (i.e. does not result in unintended consequences) and equitable (can be carried out consistently across all ITNs) [20]. Therefore, careful consideration will be required to develop a suitable approach to assessment [10]. It is likely that such an approach will include a range of methods with simulation and work-place based assessments supplemented by other approaches such as portfolios, and other forms of peer feedback. This is an important area of future research, and must be addressed if there is to be widespread adoption of competency-based approaches to teaching clinical procedures, as well as the training of junior doctors more generally [10].

There are a number of limitations that of this study. Firstly, there was likely a Hawthorne effect in the performance of the procedural skills. Therefore, the assessment should be considered to be the optimal performance of the procedures by the participants. Secondly, although steps in the procedures were derived through a combination of the clinical experience of subject matter experts, and a review of existing guidelines and protocols, a rigorous assessment of the reliability and validity of the steps in the procedures were not carried out. Thirdly, there was only one assessor at each station, so there was no assessment of the reliability of assessment. Fourthly, the assessment of performance at the first assessment was carried out before additional training was received in the procedures delivered during the boot camp completed between graduating from medical school and commencing internship. Therefore, it is not known how much of the improvements can be attributed to the training that was received during boot camp, and how much can be attributed to informal teaching and experience in the clinical setting. Fifthly, we only assessed the performance of a relatively small number of interns from one intern training network, with only one observation completed at each assessment. Therefore, this may lead to questions about the generalisability of the findings. Although the participants were drawn from one intern training network, the participants included graduates from all of the medical schools in Ireland. Therefore, we believe that the findings would be similar if performance data was collected at other intern training networks in Ireland. Finally, it may be that the COVID-19 pandemic has negatively impacted the opportunities to practice the clinical procedures during medical school, as compared to the amount of practice that is normally achieved. This is certainly possible, given that the last 18 months of the participants’ time in medical school took place during the pandemic.

## Conclusions

It is important that junior doctors can safely perform basic clinical skills. The large improvement in performance across the two assessments is encouraging. However, there is a need to address the variability in performance on graduation from medical school, and to ensure that any assessment of proficiency is not only reliant on self-report.

## Data Availability

Data is available on request from the corresponding author.
